# Raising rare disease awareness using red flags, role play simulation and patient educators: results of a novel educational workshop on Raynaud phenomenon and systemic sclerosis

**DOI:** 10.1186/s13023-020-01439-z

**Published:** 2020-06-23

**Authors:** S. Sanges, M.-M. Farhat, M. Assaraf, J. Galland, E. Rivière, C. Roubille, M. Lambert, C. Yelnik, H. Maillard, V. Sobanski, G. Lefèvre, D. Launay, S. Morell-Dubois, E. Hachulla

**Affiliations:** 1 Centre de Simulation PRESAGE, Univ. Lille, UFR Médecine, F-59000 Lille, France; 2 Univ. Lille, Inserm, CHU Lille, U1286 - INFINITE - Institute for Translational Research in Inflammation, F-59000 Lille, France; 3 Département de Médecine Interne et Immunologie Clinique, CHU Lille, F-59037 Lille Cedex, France; 4 Centre National de Référence des Maladies Auto-Immunes Systémiques Rares du Nord et Nord-Oust de France (CeRAINO), F-59000 Lille, France; 5 Health Care Provider of the European Reference Network on Rare Connective Tissue and Musculoskeletal Diseases Network (ReCONNET), Lille, France; 6 Service de médecine interne, Hôpital Lariboisière, Assistance Publique – Hôpitaux de Paris, F-75010 Paris, France; 7 Université de Paris Diderot, F-75010 Paris, France; 8 Service de médecine interne et maladies infectieuses, CHU de Bordeaux, F-33600 Pessac, France; 9 Centre de simulation SimBA-S de Bordeaux, CHU de Bordeaux et Université de Bordeaux, F-33000 Bordeaux, France; 10 Département de médecine interne, CHU de Montpellier, hôpital Lapeyronie, 371, avenue du Doyen Gaston Giraud, F-34295 Montpellier, France; 11 PhyMedExp, University of Montpellier, INSERM U1046, CNRS UMR 9214, Cedex 5 Montpellier, France; 12 CHU Lille, Institut d’Immunologie, F-59000 Lille, France

**Keywords:** Rare diseases, Systemic sclerosis, Raynaud phenomenon, Simulation, Role play, Simulated patients, Patient educators, Medical education, Big data

## Abstract

**Background:**

As lack of awareness of rare diseases (RDs) among healthcare professionals results in delayed diagnoses, there is a need for a more efficient approach to RD training during academic education. We designed an experimental workshop that used role-play simulation with patient educators and focused on teaching “red flags” that should raise the suspicion of an RD when faced with a patient with frequently encountered symptoms. Our objective was to report our experience, and to assess the improvement in learners’ knowledge and the satisfaction levels of the participants.

**Results:**

The workshop consisted of 2 simulated consultations that both started with the same frequent symptom (Raynaud phenomenon, RP) but led to different diagnoses: a frequent condition (idiopathic RP) and an RD (systemic sclerosis, SSc). In the second simulated consultation, the role of the patient was played by a patient educator with SSc. By juxtaposing 2 seemingly similar situations, the training particularly highlighted the elements that help differentiate SSc from idiopathic RP.

When answering a clinical case exam about RP and SSc, students that had participated in the workshop had a higher mean mark than those who had not (14 ± 3.7 vs 9.6 ± 5.5 points out of 20, *p* = 0.001).

Participants mostly felt “very satisfied” with this training (94%), and “more comfortable” about managing idiopathic RP and SSc (100%). They considered the workshop “not very stressful” and “very formative” (both 71%). When asked about the strengths of this training, they mentioned the benefits of being put in an immersive situation, allowing a better acquisition of practical skills and a more interactive exchange with teachers, as well as the confrontation with a real patient, leading to a better retention of semiological findings and associating a relational component with this experience.

**Conclusions:**

Through the use of innovative educational methods, such as role-play simulation and patient educators, and by focusing on teaching “red flags”, our workshop successfully improved RP and SSc learning in a way that satisfied students. By modifying the workshop’s scenarios, its template can readily be applied to other clinical situations, making it an interesting tool to teach other RDs.

## Background

The European Union considers a disease to be rare when it affects not more than 5 persons in 10.000 [1]. Although each of an estimated 6000 rare diseases (RDs) is individually uncommon, these conditions collectively affect between 27 and 36 million Europeans, making RDs a public health issue [1, 2]. One of the main challenges faced by RD patients is the “diagnostic odyssey” [3]: a survey performed in 17 European countries revealed that 25% of RD patients experienced a diagnostic delay of 5 to 30 years; and 40% of them initially received erroneous diagnoses and inappropriate treatments [4, 5]. This quest for a diagnosis is a burden with medical, psychosocial and economic consequences, some of which are severe and/or potentially avoidable.

Lack of RD awareness and insufficient knowledge among healthcare professionals is usually considered one of the main factors contributing to the diagnostic odyssey [5]. Previous studies showed that RD knowledge was frequently rated as substandard by physicians from different specialties, especially general practitioners [6–8]. Most of them felt that their academic training was insufficient and not useful for diagnosing RDs in daily practice [7–11]. Similarly, several surveys performed in different European countries consistently confirmed a poor knowledge of RDs among medical students, irrespective of the year of study [12–14]. Overall, this highlights an unmet need for a more efficient approach to RD training during academic education [15–17].

Healthcare simulation is an “educational technique that creates a situation or environment to allow persons to experience a representation of a real event for the purpose of practice, learning, evaluation, testing, or to gain understanding of systems or human actions” [18]. Simulation-based learning has proved effective in improving knowledge, skills, and behaviours of healthcare professionals [19]. Different simulation techniques exist; among them, role play is the preferred method to teach clinical and diagnostic skills to healthcare students [20–23]. Role play requires the participation of a facilitator to play the role of the patient, who can be either a trained healthy individual, called a *simulated patient* (e.g. actor, physician or peer learner), or a “real” patient, called a *patient educator* [24–26]. Although simulation can provide the opportunity to expose learners to uncommon situations (e.g. unusual complications of frequent procedures or activities, disaster medicine [27–30]), it has to our knowledge never been used to teach RD to medical students [31].

We thus imagined a novel approach to teach RD to medical students. Using role-play simulation with patient educators, we designed an experimental workshop that focused on teaching a few targeted signs or symptoms that should raise the suspicion of an RD, which we called “red flags”, rather than detailing the RD itself. To achieve this goal, we placed the learners in 2 different simulated situations that both started with the same frequent symptom (Raynaud phenomenon, RP) but led to different diagnoses: a frequent condition (idiopathic RP) and an RD (systemic sclerosis, SSc).

The aim of our study was to report our experience, evaluate the efficacy of the workshop in improving learners’ knowledge of RP and SSc and assess the satisfaction levels of the participants.

## Methods

### Study population

The workshop was offered to Lille University medical students enrolled in our “Rare Systemic Diseases” optional course. Enrolment in this course was proposed on a voluntary basis to the 536 students who, in 2018–2019, were in their fourth year of medical school (first year of clinical training in the French MD curriculum).

### Role-play workshop

The workshop was included within the syllabus of our “Rare Systemic Diseases” course. It was delivered during a dedicated half-day class within the Lille University Medical School Simulation Centre “PRESAGE”. Learners had attended a 2-h classroom lecture on RP and SSc the previous week.

The workshop consisted of two 30-min role-play stations. Learners were divided into groups of 4 or 5 persons. On each station, 2 learners were actors and played the role of physicians within the simulation; the 2 (or 3) others were observers and watched the simulation from a live video feed in the debriefing room. After a short briefing, learners took part in a 15-min role play and then re-joined the rest of the group for a 15-min debriefing with supervisors who had expertise in RP and SSc and who were trained in simulation teaching (SS, SMD). To make the situations more realistic, the learners were not informed of the final diagnosis when tackling a role-play station.

The first station simulated the case of a 26-year-old woman referred by her general practitioner for suspected RP. During the briefing, learners were instructed to perform clinical history taking and physical examination of the patient, to formulate relevant diagnosis hypotheses and to prescribe any additional examinations they considered necessary. The simulated patient was portrayed by trained physicians with expertise in RP (MMF, MA), who were briefed on the role so as to provide answers consistent with idiopathic RP. By the end of the station, learners should suspect the diagnosis of idiopathic RP. The debriefing focused on the learners’ ability to diagnose RP from other acrosyndromes (emphasising the key semiological features reported by the simulated patient), and to differentiate idiopathic from secondary RP (identifying important “red flags”, such as asymmetric attacks, thumb involvement, late onset, pulse abolition, vascular bruit, abnormal Allen’s and Roos’s tests, signs of digital ischaemia).

The second station simulated the case of a 56-year-old woman referred by her general practitioner for RP with occurrence of digital ulcers. During the briefing, learners received the same instructions as before. The role of the patient role was played by a patient educator with SSc (*see below*). By the end of the station, learners should suspect the diagnosis of secondary RP due to SSc. The debriefing focused on the learners’ ability to establish the RP as secondary (using learning acquired from the previous station), and to suspect the diagnosis of SSc (identifying major “red flags”, such as skin sclerosis, telangiectasias, calcinosis cutis, digital ulcers and pitting scars, signs of organic microangiopathy and pulmonary crackles).

**Fig. 1:**
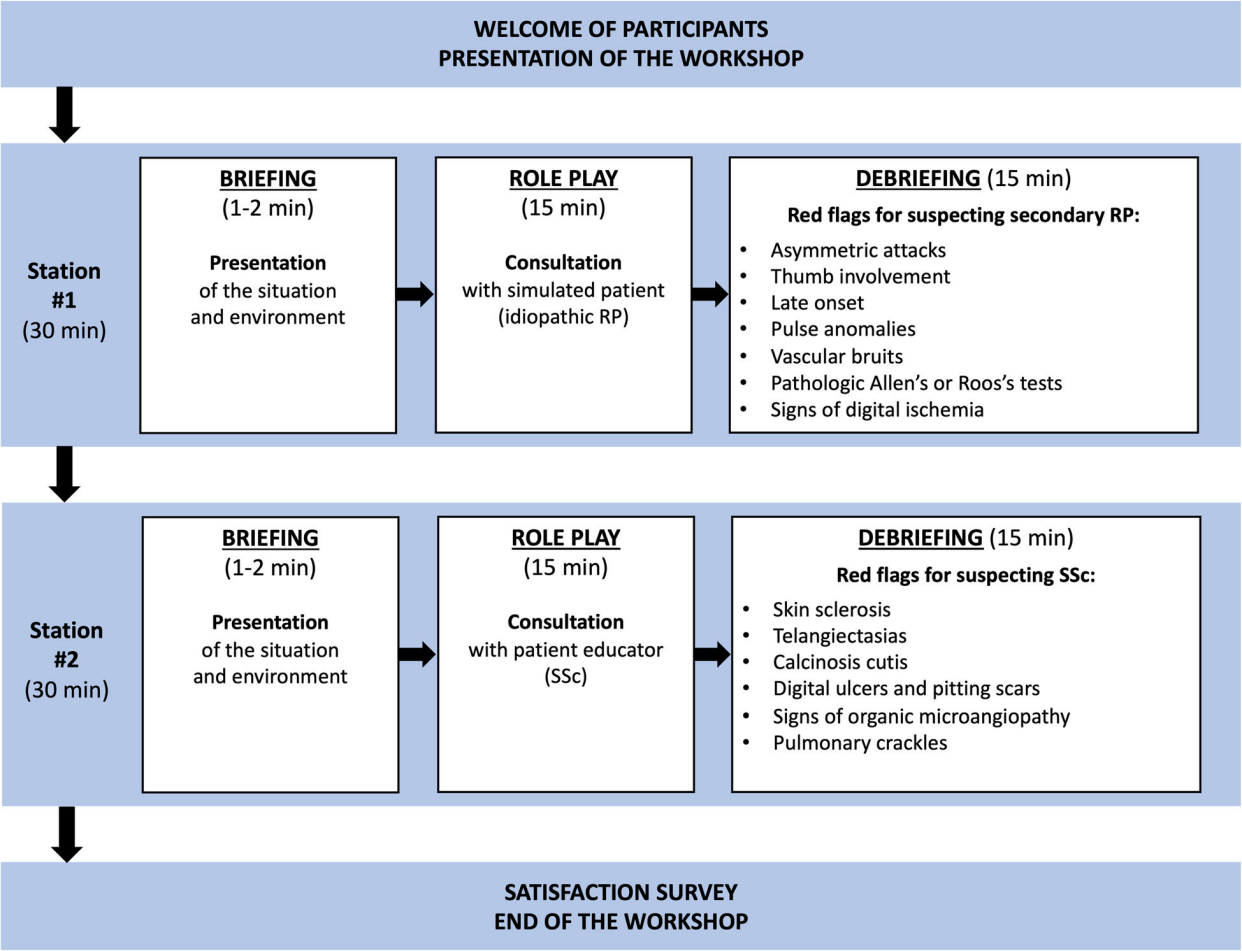
Detailed description of the workshop

The workshop sequence is summarized in Fig. 1. Briefing instructions, competence scoring grids, key debriefing elements, scenarios and character datasheets are provided in Supplemental File 1.

### Patient educator

The second station included the participation of an SSc patient educator followed in our department. She was carefully selected among other candidates based on her personal history, resilient character and settled relationship with her disease. When invited to participate, she was assured that her refusal would not interfere with her usual management. After accepting the proposition and giving her consent, she received a short informal training on the principles of simulation teaching (especially how to provide positive feedback) and the nature of her involvement (details of her part in the role play, participation in the debriefing) [32].

On the day of the workshop, the patient educator was regularly reminded that she could withdraw her participation at any time during the training for any reason. The station was jointly facilitated by her usual physician (SMD), in order to create a familiar and safe environment. She participated in the debriefing, during which she provided valuable feedback based on her own experiences as a patient.

### Satisfaction survey

At the end of this half-day class, the learners were asked to complete a satisfaction questionnaire (Supplemental File 2). The questionnaire was filled in anonymously and comprised 4 multiple-choice questions, 9 4-point Likert-scale questions and 2 open-ended questions. Optionally, learners could also provide additional comments in a free-text section of the survey.

### Final exam

The “Rare Systemic Diseases” course was sanctioned by a written exam at the end of the semester, 3 months after the workshop. The exam consisted of 2 clinical cases with 5 open-ended questions each (Supplemental File 3): one case of RP with digital ulcers revealing SSc; one case of chronic hypereosinophilia (taught in another course from the curriculum). Both these exam cases had previously been used to sanction the course for the 2014–2015 class of medical students (*n* = 41). The syllabus followed by these students was identical to that of the 2018–2019 class, except for the role-play workshop.

In order to have homogeneous corrections for both classes of students, the exam cases were marked by the same correctors (GL and DL) using identical scoring grids. To limit potential bias, these correctors were not reminded of the marks obtained by the 2014–2015 students when they corrected the 2018–2019 students’ exam papers and they were not involved in the design and facilitation of the workshop. The marks for each case were compared between the two classes using Student’s *t*-test.

## Results

A total of 21 students participated in the workshop and took the final exam; 17 of them agreed to complete the workshop satisfaction questionnaire.

### Learners’ previous experience

Before the training, 6 of the learners (35%) had previously been involved in the management of patients with RP and SSc (these students had completed a clinical rotation in our Department); and 14 (82%) had participated in an educational role play in the past. During the workshop, only 1 learner was an observer on both stations; all the others (94%) acted in the simulation in one of the stations.

### Final exam marks

**Fig. 2:**
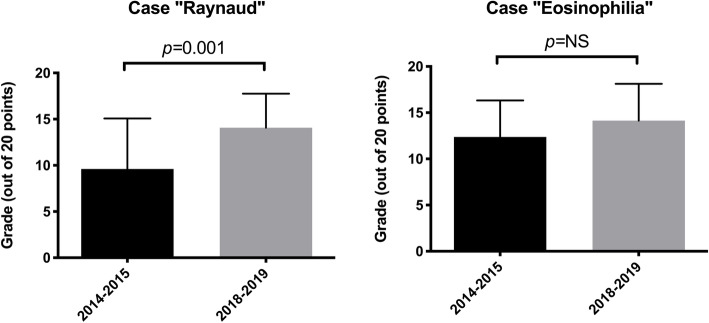
Comparison of the mean mark obtained on each exam case by the 2014–2015 and the 2018–2019 classes of medical students

To try and determine whether the workshop actually improved learning, the final exam of the course contained a clinical case of RP and SSc. The mean mark obtained by the 2018–2019 students was 14 + 3.7 points (out of 20) and was significantly higher than the mean mark of the 2014–2015 students (9.6 + 5.5 points, *p* = 0.001) (Fig. 2).

As this improvement could be explained by factors other than the workshop (e.g. possible access to previous exam questions by the 2018–2019 students), the final exam of the course included another clinical case on a different topic from the curriculum (chronic eosinophilia), which had also been used for the 2014–2015 students. There was no significant difference between the mean marks of the 2 sets of students on this exam question (14 + 4.0 points for 2018–2019 students vs 12 + 3.9 points for 2014–2015 students; *p =* 0.08), suggesting that the improvement noted on the “Raynaud” case cannot be entirely explained by prior knowledge of the exam questions and could be related to the role-play training (Fig. 2).

### Satisfaction survey

**Fig. 3:**
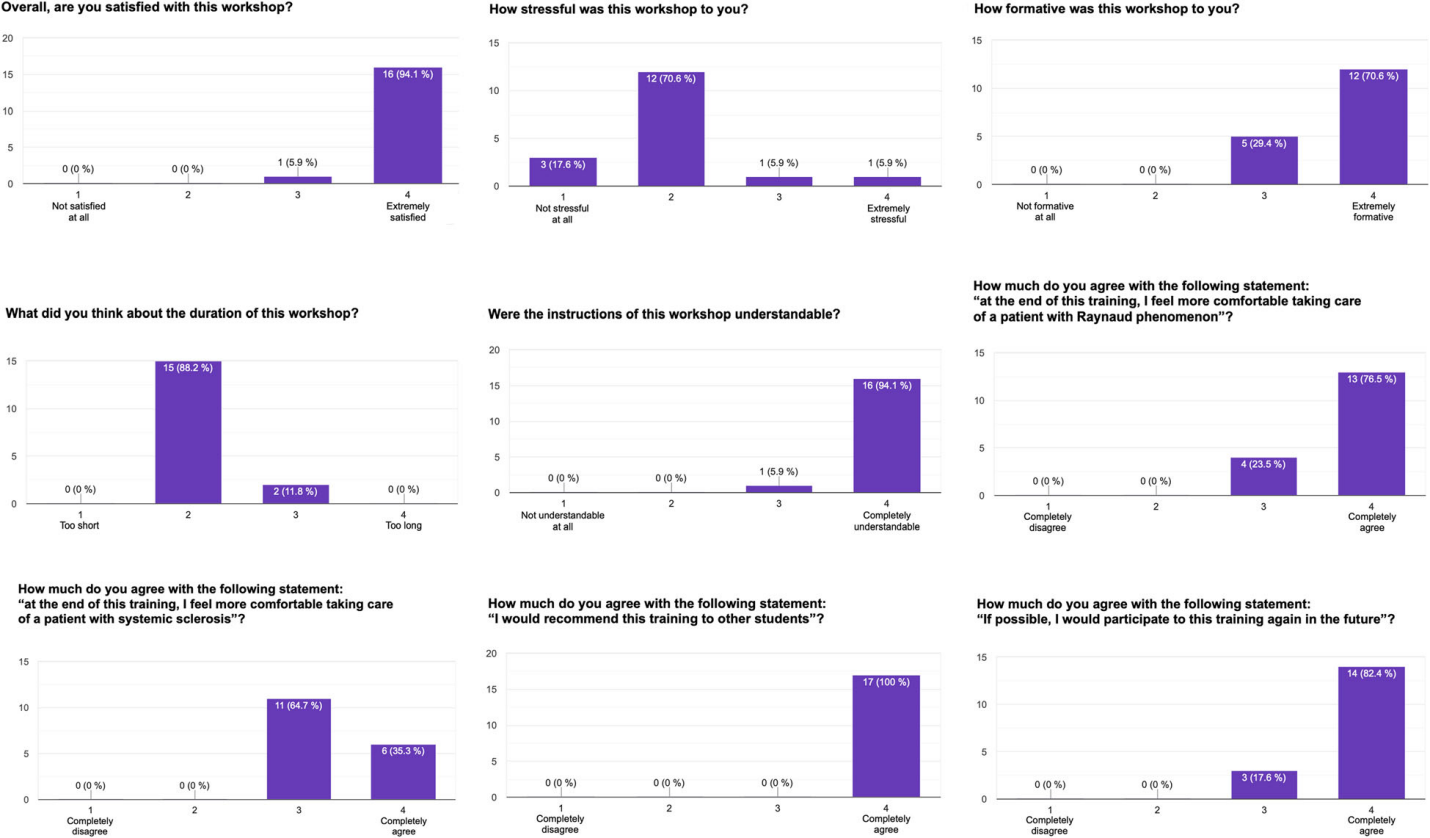
Participants’ answers to the workshop satisfaction survey

Overall, 16 learners (94%) reported they were “extremely satisfied” with this training (Fig. 3). Most of them considered the workshop “not very stressful” (71%) or “not stressful at all” (18%). All learners felt the training was “very formative” (29%) or “extremely formative” (71%). Fifteen learners (88%) considered its duration “a little too short”, with only 2 (12%) considering it “a little too long”. Almost all of them (94%) felt the instructions they had received during the training were “completely understandable”.

After participating in this workshop, all students felt “a little” (24%) or “much more comfortable” (76%) about managing patients with idiopathic RP; and “a little” (65%) or “much more comfortable” (35%) about managing patients with SSc. All of them would strongly recommend this workshop to other students, and most of them (82%) agreed that they would willingly participate in this training again in the future.

### Questions about the workshop’s strengths and limitations

**Fig. 4:**
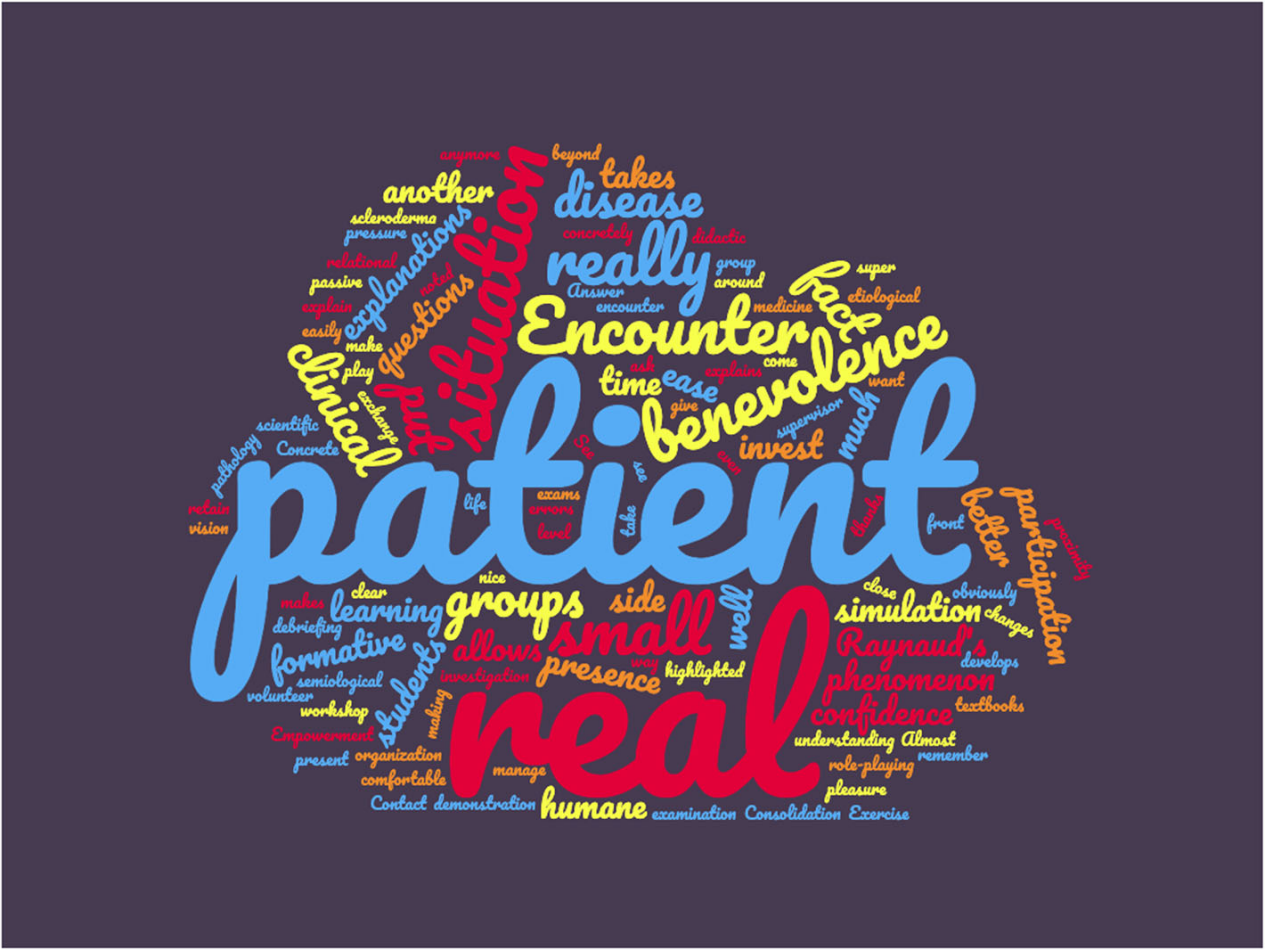
Word cloud depicting the strengths of the workshop as reported by the participants (translated from French) *Legend:* The size of each word is proportional to the frequency of its occurrence in the text of the answers provided by the learners. This picture was created using the website *nuagesdemots.fr*

Learners were asked about the strengths of this training in an open-ended question. In their answers, most of them highlighted the benefits of being placed in an immersive situation, which allowed for a better acquisition of practical skills (especially the physical examination) and a more interactive exchange with the teachers. All of them praised the encounter with a “real” patient, which allowed for a better retention of semiological findings and included a humane element in this experience. “Patient” and “real” were the words that recurred most frequently in learners’ answers (Fig. 4).

Students were then questioned about the limitations of this workshop. They reported very few weak points, which mainly concerned its short duration and the stress induced by being observed during the simulation. Most of the students (65%) reported no weaknesses at all.

## Discussion

We report here our experience with a novel approach to teaching medical students about RDs. This experimental workshop includes several original features: (1) it focuses on teaching the “red flags” that should trigger the suspicion of an RD, rather than on detailing the RD itself; (2) it uses new educational tools such as role-play simulation and patient educators; (3) it improves students’ knowledge of RP and SSc when added to conventional classroom lectures; (4) it achieves high levels of satisfaction among students.

Several studies carried out in European countries, the United States and Australia have consistently found that first-line healthcare professionals felt insufficiently informed about RDs and considered their academic training on the subject was inadequate [6–11]. Hence, the need for a change in the way we teach RDs was acknowledged by the European Rare Disease Action (RD-Action) group and the 3rd French National Plan for Rare Diseases [15–17].

### Change what we teach: adding a focus on “red flags”

Traditionally, courses on RDs have aimed at teaching medical students details of various specific conditions. These courses are usually conducted with a disease-centred approach and try to convey an overall picture of the condition, often enumerating patients’ clinical, radiological and biological characteristics [7]. We feel it would be appropriate to challenge this traditional approach and are therefore suggesting that RD courses should more actively aim to teach a “culture of doubt”, by providing medical students with a targeted list of signs and symptoms (“red flags”) that should raise suspicion of an RD in specific situations. This was previously acknowledged by several RD experts interviewed by Vandeborne et al., who suggested adopting a more patient-centred approach and focusing on teaching RD “red flags” to first-line healthcare professionals [7]. A survey performed among US clinicians supported this view, as 93.2% of primary care physicians and 85.6% of specialists agreed they should be trained about symptoms that may be indicative of a rare disorder [8]. We believe that our workshop is a successful example of this shift in the educational paradigm. RD education based on “red flags” will also become more relevant when clinical decision support algorithms based on artificial intelligence and big data are deployed in the field of RD [33].

Efforts to incorporate “red flags” teaching in RD courses have previously been reported. In Germany, Giehl et al. created the Academy for Further Medical Training on Rare Diseases (FAKSE), an organization that aims to improve RD awareness among healthcare professionals [34]: through video-based lectures and case discussions with experts, FAKSE provides them with “red flags” for better RD recognition. In Poland, Kopec et al. established an educational programme on RD for medical students that includes 10 different topics, one of which is specifically dedicated to symptoms suggesting rare conditions [35].

The structure of our workshop is quite interesting in the way it helps to highlight RD “red flags” for learners: by juxtaposing 2 clinical situations that start with the same frequent symptom but end with 2 different diagnoses (a common disorder and an RD), it brings out the elements that help differentiate SSc from idiopathic RP. This configuration can be used as a template for other situations (e.g., diarrhoea and inflammatory bowel diseases, sicca syndrome and Sjögren disease, etc.), making it an interesting educational tool for teaching any RD. This workshop can also be adapted for students training for other healthcare professions (nurses, pharmacists, etc.).

### Change how we teach: promoting educational innovation in RD

The past decades have witnessed the development of numerous new educational methods based on active learning, some of which have been tested in the field of RDs. For instance, Byrne et al. developed an RD module for medical students that was sanctioned in an innovative way [36]: first, the students had to complete a reflective learning journal at regular intervals during the semester; second, they had to prepare an information pamphlet for medical professionals detailing key elements of an RD of their choice. Learners reacted positively to this initiative [36]. As another example, Jerrentrup et al. designed a seminar using video excerpts from the TV show “House MD” as a starting point to teach RD and diagnostic strategies [37]. Several RD organizations have also helped foster medical student associations that promote RD awareness and educational endeavours [38, 39].

To our knowledge however, simulation-based learning has never been reported for RD teaching. In their policy brief for the RD-Action group, Severin et al. listed several actions that should be undertaken to improve educational programmes and training for healthcare professionals [15, 16]. One of these actions was to stimulate practical clinical training in centres of expertise, by highlighting the importance of immersive and experiential learning for RD teaching. Furthermore, the 3rd French National Plan for Rare Diseases proposed that academic training on RD should be strengthened by integrating simulation modules within the syllabus of healthcare students [17]. Our workshop thus seems to adequately meet the educational needs reported by these organizations.

Although role play is particularly suited to the teaching of clinical and diagnostic skills, other simulation techniques could be used for RD training. In a position paper, Galland et al. listed several ways of incorporating simulation in the curriculum of internal medicine residents and suggested that serious games with virtual patients could be used in training on complex diagnostic consultations [31].

It is also notable that RD training has seldom included the participation of patient educators. Byrne et al. described their innovative module in which as many as 30% of lectures were given by patients or representatives of patient support organizations, an initiative that was praised by the students [36]. The RD-Action educational policy brief highlights the importance of involving patients in educational activities, stating that it allows both fruitful student-patient interactions and patient empowerment [15, 16]. The 3rd French National Plan for Rare Diseases also encourages the development of mixed training courses that bring together healthcare professionals and patients, especially through simulation [17]. Our workshop is a suitable example of the implementation of these recommendations.

Several benefits of including patient educators in role play sessions have been reported [40]: display of clinical signs that are not easily reproduced in simulation, capacity to provide feedback on interviewing skills (e.g. communication or empathy) and physical examination (e.g. pain or discomfort), ability to share their experience of the disease to teach students about its social, psychological and emotional aspects, etc. However, a word of caution must be issued about its ethical limitations: indeed, such sessions can be perceived as patient exploitation if not conducted in full collaboration with the patient educators; and they can induce psychological distress due to the repeated narration of their illness and its impact on their life [40]. Careful attention should thus be given when considering the participation of a “real” patient in an educational role play.

### Evaluate our teaching: assessing students’ satisfaction and learning

Course evaluation is fundamental for ensuring the quality of medical education [41]. It is however rarely performed, and RD courses are no exception. In order to objectively assert the benefits of our workshop, we chose to assess our students’ learning and satisfaction. Overall, the results showed that our workshop achieved high levels of approval and improved RP and SSc knowledge, making it a level 2 study according to the Kirkpatrick model of educational evaluation [42].

To our knowledge, only 2 other studies have reported an evaluation of their RD educational initiatives: Jonas et al. showed that a 30-h RD-targeted module improved the proportion of correct answers to an RD-knowledge questionnaire [12]; and Byrne et al. reported high levels of student satisfaction with their innovative workshop that included patient lectures [36]. This highlights the need for RD teaching endeavours to be more systematically evaluated in the future.

### Strengths and limitations

Our work draws strength from the original and innovative design of our training, its generic template that makes it easily transferable to other RDs and an objective demonstration of its educational benefits.

It also has some limitations. Firstly, it was developed for small groups of students: its deployment in large groups of students could be thus challenging as it would be time-consuming and demanding in human resources (requiring more teachers, simulated patients and patient educators). A possible way to circumvent this problem would be to include senior residents within the pool of teachers and simulated patients. Secondly, it would have been preferable for the control group to be learners from the same year: unfortunately, this was not possible as it would have created inequities between students at the final exam. Thirdly, as our workshop mainly teaches hands-on diagnostic skills, it would have been more suited to assess learning using a practical evaluation (such as an objective structured clinical examination [OSCE] station) rather than through written clinical case questions. This was not possible as we would not have had a control group in that setting. Fourthly, the differences observed in the marks of the 2 sets of students could be partly explained by the 2018–2019 students having had prior knowledge of the exam questions. However, since the improvement in exam mark was not observed in the “eosinophilia” case, we believe that it is more likely to have been related to the role-play workshop. Fifthly, the difference in exam mark could also have been due to disparities in the correction of the students’ exam papers. However, as the same teachers corrected both sets of exam papers, we believe that the grading was rather homogeneous between the 2 sets of students. In order to avoid motivation biases in their correction, these teachers were not reminded of the marks of the 2014–2015 students when they corrected the 2018–2019 exam papers; furthermore they were not involved in the design or facilitation of the workshop. Lastly, although our workshop seemed to enhance RP and SSc knowledge, we did not study whether this would translate into better medical practice and patient outcomes. Further longitudinal analysis would be needed to investigate this effect.

## Conclusion

By using innovative educational methods such as role-play simulation and patient educators, and by focusing on teaching “red flags”, our workshop successfully improved RP and SSc learning in a way that satisfied learners. By modifying the workshop’s scenarios, its template can readily be applied to other clinical situations, making it an interesting tool to teach other RDs.

## Supplementary information

**Additional file 1.**

**Additional file 2.**

**Additional file 3.**

## Data Availability

The datasets used and/or analysed during the current study are available from the corresponding author on reasonable request.
