# Supplementary material for: Raising rare disease awareness using red flags, role play simulation and patient educators: results of a novel educational workshop on Raynaud phenomenon and systemic sclerosis
Source: Orphanet J Rare Dis. 2020 Jun 23;15:159. doi: 10.1186/s13023-020-01439-z (PMC7310378; doi:10.1186/s13023-020-01439-z)
Supplement: Supplementary file 1 — Additional file 1. [file 13023_2020_1439_MOESM1_ESM.zip › Supplemental File 1 EN.pdf]

Complex Diagnostic Cases:
from frequent symptoms to rare diseases

Title of the workshop:
Differential diagnosis of Raynaud phenomenon

Target audience:
- Medical students

Training objectives:
After this training, the learner will be able to collect relevant data during history taking and clinical exam, as well as to prescribe first-line additional tests, in order to:
- assert the diagnosis of Raynaud phenomenon (RP)
- identify "red flags" allowing to differentiate primary and secondary RP
- identify "red flags" suggestive of systemic sclerosis (SSc)

Preliminary learnings:
- Learners must have followed rheumatology courses about RP and SSc

Workshop sequence:
The workshop includes 2 consecutive OSCE stations:
- OSCE station #1: idiopathic RP
- OSCE station #2: RP secondary to SSc

Duration of the workshop:
Each OSCE station lasts 30 minutes (total of 1 hour for the 2 stations):
- Briefing: <1 minute
- Scenario: 15 minutes
- Debriefing: 15 minutes

Participants and roles:
- 1 or 2 physician(s): played by 1 (or 2) learners
- 1 simulated patient: played by a supervisor with expertise in RP on station #1 and by SSc patient educator in station #2
- 1 supervisor-assessor: does not participate in the role play but reserves the right to intervene as a facilitator if necessary
- Other learners (maximum 3) are observers and do not participate in the role play

Environment, layout and accessories:
- Consultation room: desk, 2 chairs, examination table
- Waiting room: 1 chair
- Equipment for the physician: gown, stethoscope, blood pressure monitor, letter from the primary care physician referring the patient, blank paper sheet, prescription pad, pen
- Equipment for the patients: none

OSCE station #1

SUPERVISOR SHEET

This first OSCE station simulates a consultation with a patient with idiopathic RP. The goal of this station is to teach learners how to diagnose RP and how to distinguish primitive from secondary causes.

Briefing:

- Give the learner(s) participating in the role play the "Physician Role Sheet" and read it aloud for the other observing learners
- Give your co-supervisor playing the part of the simulated patient the "Patient Role Sheet" which she will have studied in advance

Scenario:

During the role play, please complete the following competence scoring grid:

**INTERVIEW**		
1.	Tries to confirm the diagnosis of Raynaud's phenomenon:	
	• Seeks existence of white (syncopal) and blue (cyanic) phases	❍
	• Asks if symptoms occur by attacks / are not permanent	❍
	• Asks if cold act as a trigger	❍
2.	Tries to differentiate primary and secondary causes of Raynaud's phenomenon:	
	• Asks if symptoms are uni- or bilateral / asymmetric	❍
	• Asks if thumbs are spared	❍
	• Asks age of onset of Raynaud's phenomenon	❍
	• Asks for family history of Raynaud's phenomenon	❍
3.	Search for secondary causes of Raynaud's phenomenon:	
	• Asks for medication list	❍
	• Asks for profession	❍
	• Asks for occupations (sport)	❍
	• Asks for smoking history	❍
	• Asks for illegal drugs	❍
	• Seeks symptoms of connective tissue diseases	❍
**PHYSICAL EXAM**		
4.	Perform a vascular exam:	
	• Seek pulse anomalies on all 4 limbs	❍
	• Seek vascular bruits on all 4 limbs	❍
	• Perform Allen's test	❍
	• Perform Roos's test	❍
5.	Look for signs of digital ischemia	❍
6.	Look for signs of systemic sclerosis	❍
**ADDITIONAL TESTS**		
7.	Prescribe lab tests:	
	• Complete blood count	❍
	• Acute phase reactants (ESR, CRP)	❍
	• Anti-nuclear antibodies	❍
8.	Prescribe peri-ungueal capillaroscopy	❍

Debriefing:

Here are the main points to address during the debriefing:

- Semiological features allowing to assert the diagnosis of RP
- Interview and physical exam findings ("red flags") allowing to distinguish primitive from secondary RP
- First-line additional tests to prescribe

OSCE station #1

PHYSICIAN ROLE SHEET

You are a rheumatology resident.

You receive for the first time in consultation Ms. R. P., 26 years old, referred by her primary care physician for suspicion of Raynaud's phenomenon. You will find on the desk the letter he wrote for your attention.

You have 15 minutes to:

- perform a targeted interview focusing on the problem presented by the patient
- perform a targeted physical exam
- prescribe any additional test which appear justified at the end of the consultation

Out of respect for the person participating in this workshop, you are not allowed to ask them to undress or to carry out intimate exams.

During the physical exam, explain aloud what you are doing and enunciate your findings.

The role play begins when you bring in the patient from the waiting room.

Dr John DOE
MD

GENERAL MEDICINE

HEALTH & WELLNESS CLINIC
1600 Pennsylvania Ave NW
Washington, DC 20500
Phone : 555-123-4567

REFERRAL LETTER

Dear colleague,

I would like to refer to you Ms. R. P., 26 years old, for evaluation of a suspected Raynaud's phenomenon.

Please do accept her under your care,

Best regards,

OSCE station #1

PATIENT ROLE SHEET

Patient identity:
- *Name:* R.P.
- *Gender:* female
- *Age:* 26 y.o.

Situation at the start of the scenario:
You are sitting in the waiting room.

Initial statement:
When the learner asks you the nature of your problem, you must provide the following statement:
"I am very annoyed by flashes of pain in my fingers. When that happens, my fingers change color, and it's very uncomfortable. It's been going on for a long time, but it has become more and more frequent these past few years. I talked about this with my primary care physician, who preferred to refer me to you."
No other data should be provided spontaneously if the learner does not ask explicitly.

Interview features:
RP attacks have the following characteristics:
- Duration of about 15-20 minutes
- Localized to the fingers of both hands, except the thumbs, in a symmetrical fashion
- Moderately painful
- Fingers turn white, then blue, in a stereotypical manner
- Triggered by the cold (winter)
- Onset at around the age of 13 (during puberty)
- Never had sign of digital ischemia

Answer "no" to all other question.

Physical exam features:
The physical exam performed by the learner causes you no symptom.

General and psychological presentation:
- Good general condition
- No particular psychological distress or presentation

Past medical history:
- None
- No allergy

Family history:
- *Mother:* same symptoms as the patient
- *Rest of the family:* none

Treatments:
- None

Habits:
- *Smoking:* never
- *Alcohol:* occasionally
- *Drugs:* never
- *Job:* store manager
- *Occupations:* running
- *Family:* single, no child

Questions to ask during the scenario:
- If the learner does not spontaneously prescribe additional tests by the end of the consultation, ask the following question: *"Do I need additional exams?"*
- If the learner does not mention his/her diagnostic hypotheses by the end of the consultation, ask the following question: "*What's your diagnosis, doctor?*"

OSCE station #2

SUPERVISOR SHEET

This second OSCE station simulates a consultation with a patient with RP secondary to SSc. The goal of this station is to teach learners how to identify “red flags” suggestive of SSc.

Briefing:

- Give the learner(s) participating in the role play the “Physician Role Sheet” and read it aloud for the other observing learners
- Give your co-supervisor playing the part of the simulated patient the “Patient Role Sheet” which she will have studied in advance

Scenario:

During the role play, please complete the following competence scoring grid:

**INTERVIEW**		
1.	Looks for symptoms suggestive of systemic sclerosis:	
	• Looks for skin thickening	❍
	• Looks for shortness of breath	❍
	• Looks for cough	❍
	• Looks for GERD	❍
	• Looks for abnormal bowel movements	❍
**PHYSICAL EXAM**		
2.	Looks for physical signs suggestive of systemic sclerosis:	
	• Looks for digital ulcers and pitting scars	❍
	• Looks for telangiectasias	❍
	• Looks for signs of organic microangiopathy	❍
	• Looks for calcinosis cutis	❍
	• Looks for an abnormal Allen’s test	❍
	• Looks for skin sclerosis	❍
	• Evaluate the degree of skin thickening	❍
	• Looks for pulmonary crackles	❍
	• Looks for signs of right heart failure	❍
**ADDITIONAL TESTS**		
3.	Prescribes additional tests:	
	• Nt-pro-BNP or BNP	❍
	• Peri-ungual capillaroscopy	❍
	• Chest CT-scan	❍
	• Pulmonary function tests	❍
	• Cardiac echography	❍

Debriefing:

Here are the main points to address during the debriefing:

- Interview and physical exam findings (“red flags”) suggestive of SSc
- First-line additional tests to prescribe in case of SSc

OSCE station #2

PHYSICIAN ROLE SHEET

You are a rheumatology resident.

You receive for the first time in consultation Ms. S. Sc., 56 years old, referred by her primary care physician for severe Raynaud's phenomenon. You will find on the desk the letter he wrote for your attention, along with documents he is providing you.

You have 15 minutes to:

- perform a targeted interview focusing on the problem presented by the patient
- perform a targeted physical exam
- prescribe any additional test which appear justified at the end of the consultation

Out of respect for the person participating in this workshop, you are not allowed to ask them to undress or to carry out intimate exams.

During the physical exam, explain aloud what you are doing and enunciate your findings.

The role play begins when you bring in the patient from the waiting room.

Dr John DOE
MD

GENERAL MEDICINE

HEALTH & WELLNESS CLINIC
1600 Pennsylvania Ave NW
Washington, DC 20500
Phone : 555-123-4567

REFERRAL LETTER

Dear colleague,

I would like to refer to you Ms. S. Sc., 56 years old, for evaluation of a severe Raynaud's phenomenon with occurrence of digital ulcers (see photo attached).

I did not find any drug, toxic or occupation that could explain these symptoms. I had immunological tests performed, that found positive anti-centromere antibodies.

Please do accept her under your care,

Best regards,

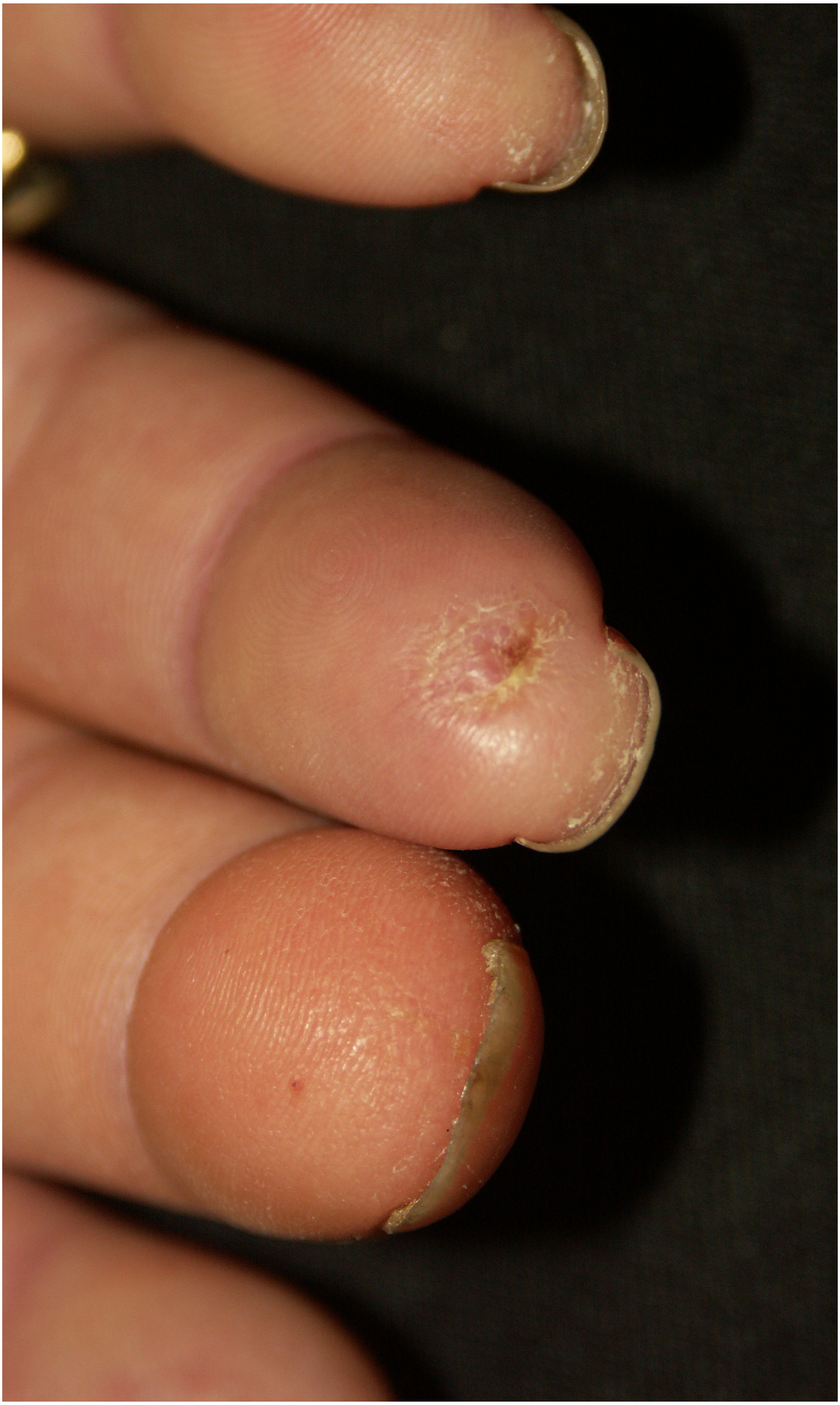

Courtesy Club Rhumatisme et Inflammation

OSCE station #2

PATIENT ROLE SHEET

Patient identity:
- *Name:* S.Sc.
- *Gender:* female
- *Age:* 56 y.o.

Situation at the start of the scenario:
You are sitting in the waiting room.

Initial statement:
When the learner asks you the nature of your problem, you must provide the following statement:
"For a few months, I have had very painful crises during which my fingers change color. My primary care physician diagnosed Raynaud's phenomenon and started to worry when I had a small loss of skin at the end of my left middle finger. That is why he referred me to you."
No other data should be provided spontaneously if the learner does not ask explicitly.

Interview features:
RP attacks have the following characteristics:
- Duration of about 30-60 minutes
- Localized to certain fingers of both hands (asymmetrical), including thumbs
- Very painful
- Fingers turn white, then blue, in a stereotypical manner
- Triggered by the cold (winter), but sometimes no obvious triggering factor
- Onset 6 months ago
- 1 digital ulcer of the left middle finger, resolving in a few weeks with local care

Other anamnestic features include:
- Feeling of puffy and tight fingers, with difficulty putting on rings
- Onset of GERD a few months before
- Onset of chronic dry cough a few months before

Answer "no" to all other question.

Physical exam features:
The physical exam performed by the learner causes you no symptom.

General and psychological presentation:
- Good general condition
- No particular psychological distress or presentation

Past medical history:
- None
- No allergy

Familial history
- None

Treatments:
- None

Habits:
- *Smoking:* never
- *Alcohol:* occasionally
- *Drugs:* never
- *Job:* cleaning person
- *Occupations:* none
- *Family:* married, 2 children

Questions to ask during the scenario:

- If the learner does not spontaneously prescribe additional tests by the end of the consultation, ask the following question: *"Do I need additional exams?"*
- Do not ask for a diagnosis if the learner does not mention it (this training is not about teaching how to break bad news). If the learner mentions the diagnosis, please react in a neutral fashion so as not to make him uncomfortable in this delicate task.
