# Supplementary material for: Raising rare disease awareness using red flags, role play simulation and patient educators: results of a novel educational workshop on Raynaud phenomenon and systemic sclerosis
Source: Orphanet J Rare Dis. 2020 Jun 23;15:159. doi: 10.1186/s13023-020-01439-z (PMC7310378; doi:10.1186/s13023-020-01439-z)
Supplement: Supplementary file 1 — Additional file 1. [file 13023_2020_1439_MOESM1_ESM.zip › Supplemental File 1 FR.pdf]

Atelier « Démarches Diagnostiques Complexes » :
du symptôme fréquent à la maladie rare

Titre de la formation :
Orientation diagnostique devant un phénomène de Raynaud

Public cible :
- Étudiants en DFASM-1, DFASM-2 ou DFASM-3

Objectifs de la formation :
A l'issue de cette formation, l'apprenant sera capable de colliger les données pertinentes d'interrogatoire et d'examen physique, et de prescrire les examens complémentaires de 1ère intention, permettant de :
- poser le diagnostic positif de phénomène de Raynaud
- identifier les drapeaux rouges permettant de distinguer le caractère primitif ou secondaire du phénomène de Raynaud
- identifier les drapeaux rouges évocateurs de sclérodermie systémique

Prérequis :
- Avoir bénéficié d'un enseignement théorique de médecine interne

Référentiel ECN :
- Item n° 237. Acrosyndromes (phénomène de Raynaud, érythermalgie, acrocyanose, engelures, ischémie digitale)

Déroulement de la formation :
La formation comprend 2 stations ECOS successives :
- Station ECOS n°1 : phénomène de Raynaud idiopathique
- Station ECOS n°2 : phénomène de Raynaud secondaire à une sclérodermie systémique

Durée de la formation :
Chaque station ECOS dure 30 minutes (soit un total d'1h pour les 2 stations) :
- Briefing : <1 minute
- Scénario : 15 minutes
- Débriefing : 15 minutes

Intervenants et rôles :
- 1 ou 2 médecin(s) : joué(s) par 1 ou 2 apprenant(s)
- 1 patient simulé : joué par un formateur (possédant une expertise dans le phénomène de Raynaud) dans l'ECOS n°1 et par un patient sclérodermique dans l'ECOS n°2
- 1 formateur-évaluateur : ne participe pas d'emblée au jeu de rôle mais se réserve le droit d'intervenir en tant que facilitateur si nécessaire
- les autres apprenants (au maximum 3) sont observateurs et n'interviennent pas dans le jeu

Environnement, aménagement et accessoires :
- Cabinet de consultation : bureau, 2 chaises, table d'examen
- Salle d'attente du cabinet : 1 chaise
- Matériel pour le médecin : blouse, stéthoscope, tensiomètre, lettre du médecin généraliste adressant le patient en consultation, feuille d'observation vierge, ordonnancier, stylo
- Matériel pour les patients : aucun

Station ECOS n°1

FICHE FORMATEUR-EVALUATEUR

Cette 1ère station ECOS simule une consultation avec une patiente présentant un phénomène de Raynaud idiopathique. L'objectif de cette station est d'enseigner à l'apprenant comment poser le diagnostic de phénomène de Raynaud et distinguer les causes primitives et secondaires.

Briefing :

- Remettre à l'apprenant (ou aux apprenants) participant au jeu de rôle la « fiche de rôle Médecin » et la lire à haute voix pour les apprenants observateurs
- Remettre à votre co-formateur jouant le rôle du patient simulé la « fiche de rôle Patient » qu'elle aura étudiée en avance

Déroulement du scénario :

Au cours du jeu de rôle, vous remplirez la grille de compétences suivante :

**INTERROGATOIRE**		
1.	Cherche à confirmer le diagnostic de phénomène de Raynaud :	
	• Recherche l'existence des phases blanche (syncopale) et bleue (cyanique)	❍
	• Recherche le caractère paroxystique des épisodes	❍
	• Recherche le caractère déclenché par le froid	❍
2.	Cherche à établir le caractère primaire ou secondaire du phénomène de Raynaud :	
	• Recherche le caractère unilatéral ou bilatéral	❍
	• Recherche une atteinte des pouces	❍
	• Recherche l'âge d'apparition du phénomène de Raynaud	❍
	• Recherche des antécédents familiaux de phénomène de Raynaud	❍
3.	Recherche des causes de phénomènes de Raynaud secondaires :	
	• Demande les prises médicamenteuses	❍
	• Demande la profession	❍
	• Demande les loisirs (sport)	❍
	• Recherche une intoxication tabagique	❍
	• Recherche une consommation de drogues illicites	❍
	• Recherche des symptômes évocateurs de connectivite	❍
**EXAMEN PHYSIQUE**		
4.	Réalise un examen vasculaire :	
	• Palpe les pouls des 4 membres	❍
	• Ausculte les axes vasculaires des 4 membres	❍
	• Réalise une manœuvre d'Allen	❍
	• Réalise une manœuvre du chandelier	❍
5.	Recherche des troubles trophiques	❍
6.	Recherche des signes physiques de sclérodermie systémique	❍
**EXAMENS COMPLEMENTAIRES**		
7.	Prescrit une biologie comprenant :	
	• NFS	❍
	• Bilan inflammatoire (VS, CRP, EPP, fibrinogène)	❍
	• Anticorps anti-nucléaires	❍
8.	Prescrit une capillaroscopie péri-unguéale	❍

Débriefing :

Les grands points à aborder lors du débriefing sont les suivants :

- Eléments sémiologiques permettant de poser le diagnostic de phénomène de Raynaud
- Eléments d'interrogatoire et d'examen physique (« drapeaux rouges ») permettant de distinguer un phénomène de Raynaud primitif et secondaire
- Examens complémentaires à prescrire en 1ère intention

<u>Station ECOS n°1</u>

FIICHE DE RÔLE MEDECIN

Vous êtes interne en médecine interne.

Vous recevez pour la première fois en consultation Mlle Mégane Renault, 26 ans, adressée en consultation par son médecin généraliste pour suspicion de phénomène de Raynaud. Vous trouverez sur le bureau la lettre qu'il a rédigée à votre attention.

Vous disposez de 15 minutes pour :

- réaliser un interrogatoire ciblé et pertinent se concentrant sur le problème présenté par la patiente
- effectuer un examen physique ciblé et pertinent
- prescrire les éventuels examens complémentaires qui vous paraissent justifiés à l'issue de la consultation

<u>Par respect pour la personne participant à cet atelier, il ne vous est pas permis de lui demander de se déshabiller, ni réaliser des examens intimes.</u>

Durant l'examen physique, expliquez à haute voix ce que vous faites et décrivez ce que vous constatez.

L'atelier commence lorsque vous allez chercher la patiente en salle d'attente et la faites entrer dans le bureau de consultation.

Docteur Alain Proviste
Diplômé de la Faculté de Médecine de Lille

MEDECINE GENERALE
----
12 Avenue du Thermomètre
59000 LILLE
Tél : 03 12 34 56 78
Fax : 03 12 34 56 79

CONSULTATIONS
SANS RENDEZ-VOUS

N° RPPS
10100123456

Lille, le 04/01/2019

Mon cher confrère,

Je vous adresse en consultation Mlle Mégane Renault, 26 ans, chez qui je suspecte un phénomène de Raynaud, pour avis spécialisé.

Vous remerciant de ce que vous ferez pour elle,

Bien confraternellement,

Station ECOS n°1

FICHE DE RÔLE PATIENT SIMULE

Identité du patient :

- *Nom :* Mégane Renault
- *Sexe :* féminin
- *Age :* 26 ans

Situation au début du scénario :
Vous patientez dans la salle d'attente du cabinet de consultation.

Déclaration initiale :
Si l'apprenant vous demande la nature du problème, vous devez lui fournir les éléments suivants :

« Je suis très embêtée par des crises de douleur dans les doigts. Quand ça se produit, mes doigts changent de couleur, et c'est assez inconfortable. Cela fait longtemps que ça dure, mais c'est de plus en plus fréquent ces dernières années. J'en ai parlé à mon médecin traitant, qui a préféré m'adresser à vous. »

Aucun autre donnée ne devra être fournie spontanément si l'apprenant ne pose pas la question.

Données cliniques de l'interrogatoire :
Les épisodes de Raynaud ont les caractéristiques suivantes :

- Durée d'environ 15-20 minutes
- Touchent les doigts des 2 mains, sauf les pouces, de façon symétrique
- Modérément douloureux
- Les doigts deviennent blancs puis bleus, de façon stéréotypée
- Déclenchés par le froid (hiver, courses au rayon surgelé)
- Apparition vers l'âge de 13 ans (lors de la puberté)
- Jamais de troubles trophiques

Répondre « non » à toutes les autres questions d'interrogatoire.

Données cliniques de l'examen physique :
L'examen physique réalisé par l'apprenant ne vous provoquera aucun symptôme.

Présentation générale et psychologique :

- Bon état général
- Pas de détresse ou de présentation psychologique particulière

Antécédents personnels :

- *Médicaux :* aucun
- *Chirurgicaux :* aucun
- *Gynéco-obstétricaux :* G0P0, cycles réguliers
- *Psychiatriques :* aucun
- *Allergiques :* aucun

Antécédents familiaux :

- *Mère :* mêmes symptômes que ceux de la patiente
- *Reste de la famille :* aucun

Traitements :

- *Médicaments habituels :* aucun
- *Médicaments ponctuels / automédication :* jamais
- *Contraception :* aucune
- *Homéo/phytothérapie :* jamais

Mode de vie :

- *Tabac :* jamais
- *Alcool :* occasionnel (festif)
- *Drogues illicites :* jamais
- *Alimentation :* normale
- *Caféine :* 1 café le matin
- *Profession :* directrice de supermarché
- *Loisirs :* 30 min de footing par semaine
- *Situation familiale :* célibataire
- *Niveau scolaire :* baccalauréat ES

Questions à poser au cours du scénario :

- Si l'apprenant ne prescrit pas spontanément d'examens complémentaires en fin de consultation, poser la question suivante : *« Est-ce qu'il faut que je fasse une prise de sang ou des examens ? »*
- Si l'apprenant ne précise pas les hypothèses diagnostiques envisagées, poser la question suivante : *« A quels diagnostics pensez-vous, docteur ? »*

Station ECOS n°2

FICHE FORMATEUR-EVALUATEUR

Cette 2ème station ECOS simule une consultation avec une patiente présentant un phénomène de Raynaud secondaire à une sclérodermie systémique. L'objectif de cette station est d'enseigner à l'apprenant comment argumenter le diagnostic de sclérodermie systémique devant un phénomène de Raynaud d'allure secondaire.

Briefing :

- Remettre à l'apprenant (ou aux apprenants) participant au jeu de rôle la « fiche de rôle Médecin » et la lire à haute voix pour les apprenants observateurs
- Remettre à votre co-formateur jouant le rôle du patient simulé la « fiche de rôle Patient » qu'elle aura étudiée en avance

Déroulement du scénario :

Au cours du jeu de rôle, vous remplirez la grille de compétences suivante :

**INTERROGATOIRE**		
1.	Recherche des symptômes évocateurs de sclérodermie systémique :	
	• Recherche une sensation d'épaississement cutané	❍
	• Recherche une dyspnée	❍
	• Recherche une toux	❍
	• Recherche un RGO	❍
	• Recherche des troubles du transit	❍
**EXAMEN PHYSIQUE**		
2.	Recherche des signes physiques évocateurs de sclérodermie systémique :	
	• Recherche (et constate, le cas échéant) la présence d'ulcérations digitales et de cicatrices pulpaires	❍
	• Recherche (et constate, le cas échéant) la présence de télangiectasies	❍
	• Recherche (et constate, le cas échéant) la présence d'une microangiopathie organique visible à l'œil nu	❍
	• Recherche (et constate, le cas échéant) la présence de calcinose sous-cutanée	❍
	• Recherche (et constate, le cas échéant) le caractère pathologique de la manœuvre d'Allen	❍
	• Recherche (et constate, le cas échéant) la présence d'une sclérose cutanée	❍
	• Apprécie l'épaisseur de la peau	❍
	• Recherche (et constate, le cas échéant) la présence de crépitants sec bi-basaux	❍
	• Recherche (et constate, le cas échéant) des signes d'insuffisance cardiaque droite	❍
**EXAMENS COMPLEMENTAIRES**		
3.	Prescrit un bilan complémentaire à l'issue de la consultation avec :	
	• Nt-pro-BNP ou BNP	❍
	• Capillaroscopie péri-unguéale	❍
	• TDM thoracique	❍
	• EFR	❍
	• ETT	❍

Débriefing :

Les grands points à aborder lors du débriefing sont les suivants :

- Eléments d'interrogatoire et d'examen physique (« drapeaux rouges ») permettant d'argumenter le diagnostic de sclérodermie systémique
- Examens complémentaires à prescrire en cas de suspicion de sclérodermie systémique

Station ECOS n°2

FICHE DE RÔLE MEDECIN

Vous êtes interne en médecine interne.

Vous recevez pour la première fois en consultation Mme Marie-Paule Dupont, 56 ans, adressée en consultation par son médecin généraliste pour phénomène de Raynaud sévère. Vous trouverez sur le bureau la lettre qu'il a rédigée à votre attention, ainsi que les éléments qu'il vous fournit.

Vous disposez de 15 minutes pour :

- réaliser un interrogatoire ciblé et pertinent se concentrant sur le problème présenté par la patiente
- effectuer un examen physique ciblé et pertinent
- prescrire les éventuels examens complémentaires qui vous paraissent justifiés à l'issue de la consultation

Par respect pour la personne participant à cet atelier, il ne vous est pas permis de lui demander de se déshabiller, ni réaliser des examens intimes.

Durant l'examen physique, expliquez à haute voix ce que vous faites et décrivez ce que vous constatez.

L'atelier commence lorsque vous allez chercher la patiente en salle d'attente et la faites entrer dans le bureau de consultation.

Docteur Alain Proviste
Diplômé de la Faculté de Médecine de Lille

MEDECINE GENERALE
----
12 Avenue du Thermomètre
59000 LILLE
Tél : 03 12 34 56 78
Fax : 03 12 34 56 79

CONSULTATIONS
SANS RENDEZ-VOUS

N° RPPS
10100123456

Lille, le 04/01/2019

Mon cher confrère,

Je vous adresse en consultation Mme Marie-Paule Dupont, 56 ans, qui présente un phénomène de Raynaud sévère qui s'est compliqué de troubles trophiques. Je vous en fournis une photo.

Je n'ai pas trouvé de facteurs médicamenteux, toxiques ou professionnels susceptibles d'expliquer ce phénomène de Raynaud d'allure secondaire. J'ai donc fait un bilan immunologique qui retrouve des anticorps anti-centromères.

Je vous l'adresse pour avis spécialisé et prise en charge.

Vous remerciant de ce que vous ferez pour elle,

Bien confraternellement,

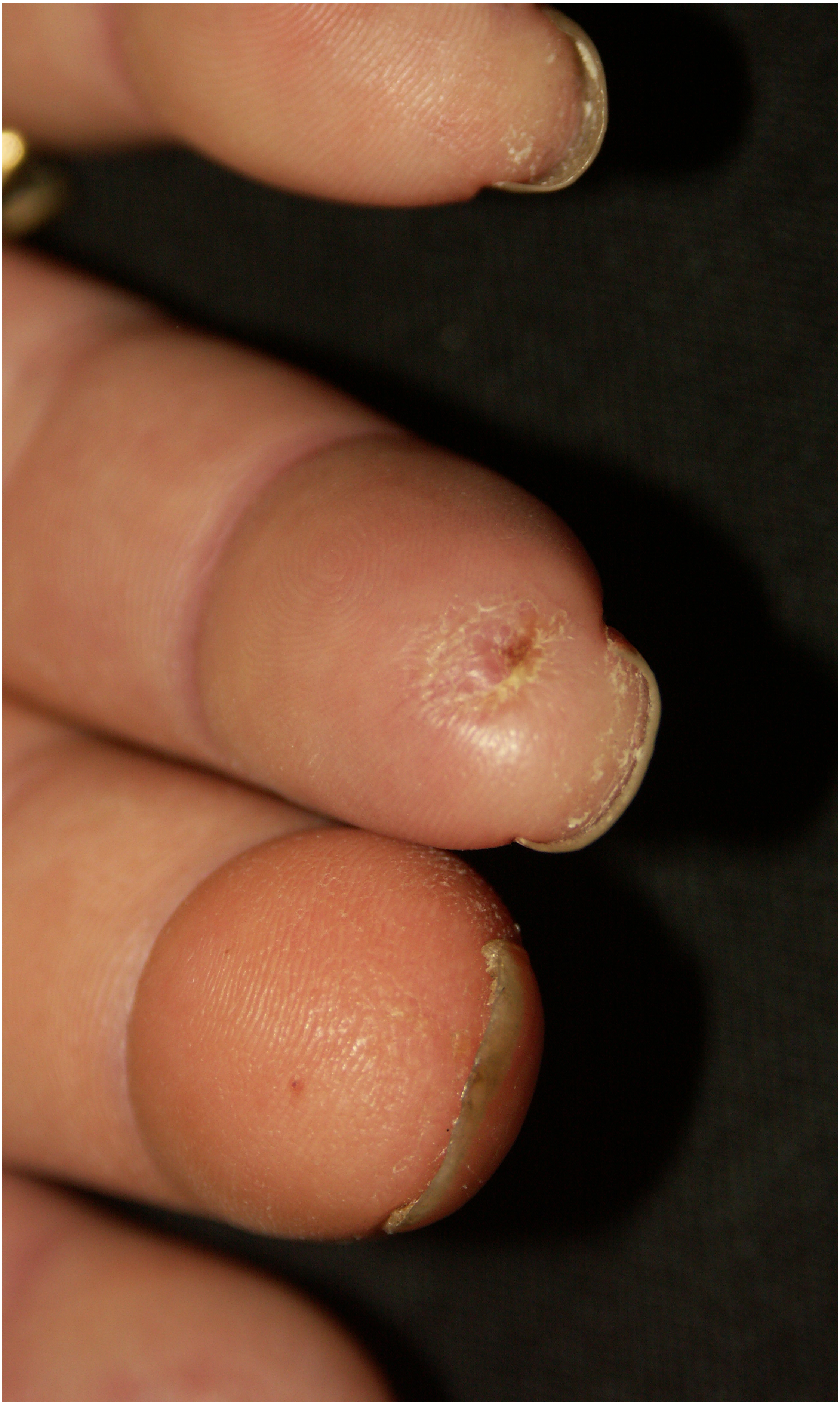

Source : Club Rhumatisme et Inflammation

Station ECOS n°2

FICHE PATIENT SIMULE

Identité du patient :
- *Nom :* Marie-Paule Dupont
- *Sexe :* féminin
- *Age :* 56 ans

Situation au début du scénario :
Vous patientez dans la salle d'attente du cabinet de consultation.

Déclaration initiale :
Si l'apprenant vous demande la nature du problème, vous devez lui fournir les éléments suivants :

« Depuis quelques mois, j'ai des crises très douloureuses au cours desquelles mes doigts changent de couleur. Mon médecin généraliste a parlé de phénomène de Raynaud et s'est inquiété quand j'ai eu une petite perte de chair au bout du majeur gauche. C'est pour cela qu'il m'adresse à vous. »

Aucun autre donnée ne devra être fournie spontanément si l'apprenant ne pose pas la question.

Données cliniques de l'interrogatoire :
Les épisodes de Raynaud ont les caractéristiques suivantes :
- Durée d'environ 30 à 60 minutes
- Touchent certains doigts des 2 mains (asymétrique), y compris les pouces
- Très douloureux
- Les doigts deviennent blancs puis bleus, de façon stéréotypée
- Déclenchés par le froid (hiver, courses au rayon surgelé), mais parfois pas de facteur déclenchant évident
- Apparition il y a 6 mois
- 1 épisode d'ulcère digital du majeur gauche, résolutif en quelques semaines sous soins locaux

Les autres signes d'interrogatoire sont les suivants :
- Sensation de doigts boudinés et tendus depuis quelques mois, avec difficultés à enfiler les bagues
- Apparition d'un RGO depuis quelques mois
- Apparition d'une toux sèche chronique depuis quelques mois

Répondre « non » à toutes les autres questions d'interrogatoire.

Données cliniques de l'examen physique :
L'examen physique réalisé par l'apprenant ne vous provoquera aucun symptôme.

Présentation générale et psychologique :
- Bon état général
- Pas de détresse ou de présentation psychologique particulière

Antécédents personnels :
- *Médicaux :* aucun
- *Chirurgicaux :* appendicectomie
- *Gynéco-obstétricaux :* G2P2, ménopausée
- *Psychiatriques :* aucun

Antécédents familiaux :
- Aucun

Traitements :
- *Médicaments habituels :* aucun
- *Médicaments ponctuels / automédication :* jamais
- *Traitement hormonal :* aucune
- *Homéo/phytothérapie :* jamais

Mode de vie :
- *Tabac :* jamais
- *Alcool :* occasionnel (festif)
- *Drogues illicites :* jamais
- *Alimentation :* normale
- *Caféine :* 1 café le matin
- *Profession :* femme de ménage
- *Loisirs :* aucun (pas de sport)
- *Situation familiale :* mariée, 2 enfants
- *Niveau scolaire :* brevet d'études

<u>Questions à poser au cours du scénario :</u>

- Si l'apprenant ne prescrit pas spontanément d'examens complémentaires en fin de consultation, poser la question suivante : *« Est-ce qu'il faut que je fasse une prise de sang ou des examens ? »*
- Ne pas évoquer le diagnostic si l'apprenant n'en parle pas (cet atelier n'évalue pas l'annonce du diagnostic). Si l'apprenant annonce le diagnostic, réagir de façon neutre afin de ne pas le mettre en difficulté dans cette tâche difficile.
