# Supplementary material for: Raising rare disease awareness using red flags, role play simulation and patient educators: results of a novel educational workshop on Raynaud phenomenon and systemic sclerosis
Source: Orphanet J Rare Dis. 2020 Jun 23;15:159. doi: 10.1186/s13023-020-01439-z (PMC7310378; doi:10.1186/s13023-020-01439-z)
Supplement: Supplementary file 2 — Additional file 2. [file 13023_2020_1439_MOESM2_ESM.pdf]

Satisfaction survey submitted to the participants at the end of the workshop.

1. Before this training, had you ever met a patient with Raynaud phenomenon?

❍ Yes ❍ No ❍ Other: ______________________

2. Before this training, had you ever met a patient with systemic sclerosis?

❍ Yes ❍ No ❍ Other: ______________________

3. Before this training, had you ever participated to an educational role play?

❍ Yes ❍ No ❍ Other: ______________________

4. During this training, were you an actor or an observer?

❍ Actor ❍ Observer ❍ Other: ______________________

5. Overall, are you satisfied with this workshop?

	1	2	3	4	
Not satisfied at all	❍	❍	❍	❍	Extremely satisfied

6. How stressful was this workshop to you?

	1	2	3	4	
Not stressful at all	❍	❍	❍	❍	Extremely stressful

7. How formative was this workshop to you?

	1	2	3	4	
Not formative at all	❍	❍	❍	❍	Extremely formative

8. What did you think about the duration of this workshop?

	1	2	3	4	
Too short	❍	❍	❍	❍	Too long

9. Were the instructions of this workshop understandable?

	1	2	3	4	
Not understandable at all	❍	❍	❍	❍	Completely understandable

10. How much do you agree with the following statement: “at the end of this training, I feel more comfortable taking care of a patient with Raynaud phenomenon”?

	1	2	3	4	
Completely disagree	❍	❍	❍	❍	Completely agree

11. How much do you agree with the following statement: “at the end of this training, I feel more comfortable taking care of a patient with systemic sclerosis”?

	1	2	3	4	
Completely disagree	❍	❍	❍	❍	Completely agree

12. How much do you agree with the following statement: “I would recommend this training to other students”?

	1	2	3	4	
Completely disagree	❍	❍	❍	❍	Completely agree

13. How much do you agree with the following statement: "If possible, I would participate to this training again in the future"?<br>1 2 3 4<br>Completely disagree ❍ ❍ ❍ ❍ Completely agree
14. In your opinion, what were the strengths of this training?<br>____________________<br>____________________<br>____________________<br>____________________
15. In your opinion, what were the weaknesses of this training?<br>____________________<br>____________________<br>____________________<br>____________________
Do you have any extra comments on this training? (optional)<br>____________________<br>____________________<br>____________________<br>____________________
