# Supplementary material for: Raising rare disease awareness using red flags, role play simulation and patient educators: results of a novel educational workshop on Raynaud phenomenon and systemic sclerosis
Source: Orphanet J Rare Dis. 2020 Jun 23;15:159. doi: 10.1186/s13023-020-01439-z (PMC7310378; doi:10.1186/s13023-020-01439-z)
Supplement: Supplementary file 3 — Additional file 3. [file 13023_2020_1439_MOESM3_ESM.pdf]

Final Exam – Clinical Case #1 – Raynaud Phenomenon

A 42-year-old patient is referred to your practice on cold day of January for a pain in her 4th right digit. The pain started 3 weeks ago and gradually increased to become intolerable. Further interrogation revealed that she has been complaining of Raynaud phenomenon for a few years. The digit appears cyanotic with signs of ischemia and necrosis on clinical examination.

Question 1: What are the most relevant clues to look for when interviewing the patient in order to determine the cause of this acrosyndrome?

Question 2: What are the most relevant clues to look for when performing clinical examination in order to determine the cause of this acrosyndrome?

Question 3: Nailfold capillaroscopy revealed signs of organic microangiopathy. What is your first diagnostic hypothesis?

Question 4: Which biological tests do you perform in order to confirm your hypothesis?

Question 5: The suspected diagnosis is confirmed. What additional tests do you prescribe in order to complete the initial work-up of the disease?

Final Exam – Clinical Case #2 – Eosinophilia

A 63-year-old patient is referred to your practice for chronic eosinophilia that started 2 years ago. The eosinophil blood count progressively increased to reach 1 G/L on the last CBC.

Question 1: What are the most frequent causes of chronic eosinophilia to look for when interviewing the patient?

Frequent causes of chronic eosinophilia are ruled out. Review of symptoms does not reveal any complaint. Physical exam reveals a moderate spleen enlargement.
Addition laboratory tests are performed. CBC finds a persistent elevation of eosinophil levels at 2.2 G/L and a non-regenerative normocytic anemia. Kidney and liver functions are normal; and acute phase reactants within normal ranges. HIV serology is negative. Total IgE levels are normal. A chest and abdomen CT-scan is performed and reveals an 18-cm splenomegaly without lymph node enlargement.
When retrieving previous CBC, you notice that corticosteroids prescribed for upper respiratory tract infection did not modify the eosinophil blood count.

Question 2: Identify in this observation the elements suggesting clonal eosinophilia. What other simple biological anomalies could you look for to support this hypothesis?

Question 3: What additional tests will you prescribe to formally confirm this hypothesis?

Question 4: What additional tests will you prescribe to identify organ involvements related to the chronic eosinophilia?

Question 5: You have identified the most frequent mutation associated with isolated clonal eosinophilia. What treatment will you start?
